# Optimization of orthogonal adaptive waveform design in presence of compound Gaussian clutter for MIMO radar

**DOI:** 10.1186/s40064-015-1501-x

**Published:** 2015-12-22

**Authors:** B. Roja Reddy, M. Uttarakumari

**Affiliations:** R V C E, Bangalore, India

**Keywords:** Orthogonal optimal waveform, Adaptive filter, Secondary data, Signal to interference noise ratio (SINR), Generalized likelihood ratio test (GLRT)

## Abstract

In this paper, an adaptive algorithm is proposed to develop an orthogonally optimized waveforms with good correlation properties that are suitable for the detection of target in the presence of strong clutter. The joint optimization both at the transmitter and receiver is adapted based on the secondary data and clutter to maximize signal to interference noise ratio (SINR) with target and clutter knowledge. The result shows good correlation properties and better SINR and signal to clutter ratio (SCR) compared to the existing iterative algorithm. The proposed algorithm also shows improved detection even for lower SCR when implemented with GLRT.

## Background

Multiple input and multiple output systems (MIMO) radiate multiple probing signals through it their transmit antennas and receive multiple coded waveforms from multiple locations. MIMO radar systems have many advantages including high resolution target detection/estimation (Haimovich et al. 2008), significantly improved parameter identifiability (Li et al. 2007).

The performance of the transmitted waveforms is judged by their correlation properties (Deng et al. 2004; Liu et al. 2007, 2008). The waveforms with good autocorrelation properties provide high range resolution and good crosscorrelation helps in multiple target return separability. So, there is a need to design MIMO radar waveforms as orthogonal pulses with low correlation properties. In literature, the orthogonal sequences are generated with low autocorrelation and crosscorrelation peak sidelobe levels using various algorithms. Deng et al. (2004) has proposed simulated annealing (SA) algorithm to optimize the frequency sequences for the development of the orthogonal discrete frequency coding waveforms frequency hopping (DFCW_FF) for netted radar systems. Liu has proposed orthogonal DFCW_FF (Liu et al. 2007) and orthogonal discrete frequency coding waveforms linear frequency modulation (DFCW_LFM) (Liu et al. 2008) using a modified genetic algorithm (MGA).

A multi-objective optimization (MOO) algorithm (Sen et al. 2013) was proposed to maximize the SINR using the orthogonal frequency division multiplexing (OFDM) radar signal with the prior knowledge of target and noise covariance. The two-stage waveform optimization (Nijsure et al. 2013) algorithm maximizes signal-to-clutter-plus-noise ratio (SCNR) for adaptive distributed MIMO radar. An optimal transmit waveform was derived by maximizing the signal-to-noise ratio (SNR) (Friedlander et al. 2006) of the transmitted signal by controlling the space–time distribution to obtain significant improvement in the detection performance. The optimization of both waveforms and the receiving filters by iterative algorithm (Chen and al 2009) maximizes signal-to-interference noise ratio (SINR). An adaptive OFDM (Sen and Glover 2012) radar signal was designed to detect a target employing spectral weights for the next transmitting waveform to maximize SNR. Adaptive MIMO radar waveform (Zhang et al. 2009) algorithm was designed to improve the target detection by maximizing the MI between the target impulse response and the received echoes and also minimize the MMSE in estimating the target impulse response. From the literature it is understood that the algorithms have considered either orthogonality or optimality for the design of waveforms but not both.

In this paper, ortho-optimal waveforms with good correlation properties that are suitable for the detection of various targets in the presence of clutter with prior knowledge of target and clutter are presented using adaptive algorithm. This algorithm is based on continuous training of the receiver and the transmit waveform on the basis of environment change to suit best the dynamic radar scene. The performance measures used in this paper are SINR, SNR and signal-to-clutter ratio (SCR).

Rest of paper is organized as follows. In “Signal model” we formulate orthogonally optimized algorithm for the DFCW waveform design in order to minimize the cost function. In “System model” we introduce system model and orthogonally adaptive optimization algorithm. Design results from the proposed algorithms are discussed in “Results”. Finally conclusions are drawn in “Conclusion”.

## Signal model

Consider MIMO radar system with N transmitting antennas, each represented by a sequence of M samples and R receiving antennas. A modified ant colony optimization algorithm (M_ACO) is used to generate orthogonal discrete frequency waveforms (DFCW) with good correlation properties. To achieve this objective, the cost function was based on peak sidelobe and integrated sidelobes level ratio is considered for minimizing objective function.

### Discrete frequency coding waveform

Discrete frequency coding (DFC) sequence is represented as {0, 1, 2, 3… M − 1} randomly. The waveform with adjacent subpulses of time duration modulated with DFC sequences is called as DFCW. Each pulse is divided into number of subpulses in the waveform which are equal to the number of code sequences. The DFCW_LFM waveform is defined as (Liu et al. 2008)

1 $${\mathbf{a}}_{{\mathbf{p}}} \left( {\mathbf{t}} \right) = \left\{ {\begin{array}{*{20}l} {\sum\nolimits_{{{\mathbf{n}} = {\mathbf{0}}}}^{{{\mathbf{M}} - {\mathbf{1}}}} {{\mathbf{e}}^{{{\mathbf{j2}}\,{\mathbf{\pi f}}_{{\mathbf{n}}}^{{\mathbf{p}}} \,{\mathbf{(t}} - {\mathbf{nT}}_{{}} {\mathbf{)}}}} \cdot {\mathbf{e}}^{{{\mathbf{j}}\,2{\varvec{\uppi}}\,{\mathbf{ kt}}^{{\mathbf{2}}}}}}}, & \quad {{\mathbf{0}} \le {\mathbf{t}} \le {\mathbf{T}}} \\ {{\mathbf{0,}}} & \quad {{\mathbf{elsewhere}}} \\ \end{array} \quad , \quad {\text{p}} = 1 , 2 ,\ldots , {\text{N}} .} \right.$$

where B is DFCW bandwidth, T is the subpulse time duration and k is the frequency slope, k = B/T. p = 1, 2,…, N. M represents number of subpulses with the coefficient sequence {m_1_,m_2_,… m_M_} with unique permutation of sequence {0,1,2,… M − 1}. The f_m_^p^ = f_o_^p^ + m ∆f is the coding frequency of mth subpulse, being the starting frequency of p-th waveform. ∆f is the frequency step. The B and T values are constant for each pulse. The grating lobes can be eliminated by the relationship between subpulse duration T, frequency step ∆f and LFM bandwidth B. The starting frequency of each LFM pulse is different. The above mentioned parameters are different for different lengths of firing sequence. The choice of BT, T ∆f and B/∆f values are crucial for the waveform design (Liu et al. 2008), as shown in Table 1. The B and T values are constant for each pulse. These BT, T ∆f and B/∆f values are selected from the Table 1 depending on the value of M which are proposed in (Liu et al. 2008) so that the grating lobes can be eliminated.

**Table 1:** Length of firing sequences and related parameters (Liu et al. 2008)

M	B·T	B/∆f	T·∆f
8	18	6	3
16	36	12	3
32	72	24	3
64	144	48	3
128	288	96	3

### Cost function

The cost function is the key parameter for the waveform optimization. The peak side-lobe ratio (PSLR) and the integrated side-lobe ratio (ISLR) determines the correlation properties. The PSLR is a ratio of the amplitude of the peak sidelobe to the main lobe and is expressed in decibels. This parameter ensures the detection of weak targets when covered by strong ones. The autocorrelation and crosscorrelation PSLR is given by

$${\text{PSLR}}_{\text{An}} = 2 0 {\text{log}}_{ 1 0} \left\{ {{ \hbox{max} }_{\text{n}} \in {\text{sidelobe}}\left| {{\mathbf{A}}\left( {{\mathbf{S}}_{\text{t}} ,\,{\text{n}}} \right)} \right|} \right\}$$

2 $${\text{PSLR}}_{\text{Cn}} = 2 0 {\text{log}}_{ 1 0} \left\{ {{ \hbox{max} }_{\text{n}} \in \left| {{\mathbf{C}}\left( {{\mathbf{S}}_{\text{t}} ,\,{\mathbf{S}}_{\text{q}} ,\,{\text{n}}} \right)} \right|} \right\}$$

where t ≠ q, t = 1, 2,… N, q = 1, 2,… N, n = 1,2,..N for PLSR_An_ and n = 1, 2, … N(N − 1) for PLSR_Cn_, **A**(S_t_, n) and **C**(S_t_, S_q_, n) are the aperiodic autocorrelation function of t-th waveform, the crosscorrelation function of t-th and q-th waveforms, respectively.

The ISLR is a ratio of sum of the energy side lobes to the energy of the main lobe in the pulse compression function. The autocorrelation and crosscorrelation ISLR is given by

$${\text{ISLR}}_{\text{An}} = 2 0 {\text{log}}_{ 1 0} \left\{ {\sum\limits_{t = 1}^{N} {{\text{Sidelobe}}\left| {{\mathbf{A}}\left( {{\mathbf{S}}_{\text{t}} ,\,{\text{n}}} \right)} \right|} } \right\}$$

3 $${\text{ISLR}}_{\text{Cn}} = 2 0 {\text{log}}_{ 1 0} \left\{ {\sum\limits_{t = 1}^{N} {\sum\limits_{q = 1}^{N} {\left| {{\mathbf{C}}\left( {{\mathbf{S}}_{\text{t}} ,\,{\mathbf{S}}_{\text{q}} ,\,{\text{n}}} \right)} \right|} } } \right\}$$

where t ≠ q, t = 1, 2,… N, q = 1, 2, … N, n = 1, 2, … 2N − 1.

The objective is to minimize the cost function (CF) and is given as (Bo et al. 2011):

4 $${\text{CF}} = \sum\limits_{{{\text{n}} = 1}}^{\text{N}} {{\text{PSLR}}_{\text{An}} } + \, \sum\limits_{{{\text{n}} = 1}}^{{{\text{N}}({\text{N}} - 1)}} {{\text{PSLR}}_{\text{Cn}} } + \sum\limits_{{{\text{n}} = 1}}^{{2{\text{N}} - 1}} {{\text{ISLR}}_{\text{An}} } + \sum\limits_{{{\text{n}} = 1}}^{{2{\text{N}} - 1}} {{\text{ISLR}}_{\text{Cn}} }$$

Subjected to power constraint ||s||^2^ ≤ 1.

## System model

The N waveforms of length M are transmitted and reflected by a target and clutter. In the receiver N × R waveforms are recovered and to further detect the target detection by a receiving filter. K (K ≥ N) secondary data vector and primary data share the same covariance structure. The covariance matrix is a trained matrix of clutter statistics for K secondary data. r_i_ and r_iK_, i = 1, 2… r, K = 1, 2 … K are primary and secondary data of the received signal, respectively. The primary data received by the radar at the i-th antenna are given by

5 $${\mathbf{r}}_{{\mathbf{i}}} = {\mathbf{S\alpha }}_{{\mathbf{i}}} + {\mathbf{Sc}}_{{\mathbf{i}}} + {\mathbf{n}}_{{\mathbf{i}}} ,\quad {\text{i}} = 1 ,\ldots {\text{R}}$$

where, ${\mathbf{S}}_{{{\mathbf{N}} \times {\mathbf{M}}}} = {\mathbf{[a}}_{{\mathbf{1}}} {\mathbf{,}} \ldots {\mathbf{,a}}_{{\mathbf{N}}} {\mathbf{]}}\; \in \;{\mathbf{C}}^{{{\text{N}} \times {\text{M}}}}$ is the transmit code matrix and ${\mathbf{a}}_{{\mathbf{n}}} = [{\mathbf{a}}_{\text{n1}} ,{\mathbf{a}}_{\text{n2}} , \ldots ,{\mathbf{a}}_{\text{NM}} ]^{\text{T}} \in {\mathbf{C}}^{\text{Nx1}}$, the transmit codeword of the antenna with M as the length of the code word where, the superscript *T* stands for the transpose of a matrix. The target scattering properties are represented by **α**_**i**_ **=** **[**α_i1_, α_i2_,… α_iN_]^T^, i = 1, … R, which is the target scattering coefficient generated randomly and complex and those of the clutter by **c**_**i**_, which is the clutter vector. An additive complex Gaussian noise vector is **n**_i_. The target scattering is given by **α** **=** **Ƙ δ(t** **−** **τ)** where τ < 2d/c, the radial span of the target is d and speed of light is c. The reflection coefficient of individual scatters is Ƙ which are generated randomly and are complex values.

Additionally, a set of K(K ≥ N) secondary data vectors is necessary to trained clutter statistics for K secondary data for the implementation of orthogonal adaptive optimization algorithm. The secondary data vectors are defined as

6 $${\mathbf{r}}_{{{\mathbf{iK}}}} = {\mathbf{Sc}}_{{{\mathbf{iK}}}} + {\mathbf{n}}_{{{\mathbf{iK}}}} ,\quad {\text{i}} = 1 ,\ldots {\text{R,}}\;{\text{K}} = 1 , 2\ldots {\text{K}}$$

where, is an additive complex Gaussian noise of secondary vector is **n**_iK_ and those of the clutter c_iK_ is the secondary clutter vector.

As the resolution of radar system increases, the clutter model no longer acts as Gaussian distribution (Fay et al. 1997; Trunk 1973; Jakeman and Pusey 1976; Gini et al. 2002; Hu et al. 2006). The model of sea clutter is a challenge to fit various distributions. The models proposed are Weibull (Fay et al. 1997), log-normal (Trunk 1973), k (Jakeman and Pusey 1976) and compound Gaussian (Gini et al. 2002) distributions. These models do not satisfactorily match to real sea clutter. The limitation of these models is due to non stationary characteristic. The Tsallis distribution (Hu et al. 2006) is used to model the sea clutter, known as K-distribution clutter. This K-distribution sea model is verified with original amplitude data of sea clutter. This is the best distribution for sea clutter (Ward 1981).

The compound Gaussian random vector, **c**_**i**_ is given as, i.e.,

7 $${\mathbf{c}}_{\text{i}} = \sqrt {{\varvec{\upalpha}}_{{\mathbf{i}}} {\varvec{\upbeta}}_{{\mathbf{i}}} } ,\quad {\text{i}} = 1 ,\ldots {\text{R}}$$

The texture **α**_**i**_ is non negative random variable and the speckle component and β_i_ is correlated complex circular Gaussian vector. The compound Gaussian clutter is sample from K-distribution.

Noise covariance matrix is given by

8 $${\mathbf{R}}_{{\mathbf{n}}} = {\text{E}}\left[ {{\mathbf{n}}_{{\mathbf{i}}} {\mathbf{n}}_{\text{i}}^{{^{\text{H}} \, }} } \right]$$

where, H is transpose conjugate of a matrix and E[.] is expectation operator.

Matched filter output at the receiver is expressed as

9 $${\mathbf{y}} = {\mathbf{h}}^{\text{H}} {\mathbf{r}} = \mathop {\underline{{{\mathbf{h}}^{\text{H}} {\mathbf{\alpha S}}}} }\limits_{\text{signal}} + \mathop {\underline{{{\mathbf{h}}^{\text{H}} {\mathbf{cS}}}} }\limits_{\text{clutter}} + \mathop {\underline{{{\mathbf{h}}^{\text{H}} {\mathbf{n}}}} }\limits_{\text{noise}}$$

where h is the impulse response of the matched filter at the receiver of size (1 × N). The matched filter output at the receiver y is of size (1 × N)

Thus, the SINR, SNR, SCR at the filter can be expressed as

10 $${\text{SINR}} = \frac{{\left\| {{\mathbf{h}}^{H} {\mathbf{\alpha S}}} \right\|^{ 2} }}{{{\text{E}}\left[ {\left\| {{\mathbf{h}}^{H} {\mathbf{cS}}} \right\|^{ 2} } \right] + {\text{E}}\left[ {\left\| {{\mathbf{h}}^{\text{H}} {\mathbf{n}}} \right\|^{ 2} } \right]}},\quad {\text{SNR}} = \frac{{\left\| {{\mathbf{h}}^{H} {\mathbf{\alpha S}}} \right\|^{ 2} }}{{{\text{E}}\left[ {\left\| {{\mathbf{h}}^{H} {\mathbf{n}}} \right\|} \right]^{2} }},\quad {\text{SCR}} = \frac{{\left\| {{\mathbf{h}}^{H} {\mathbf{\alpha S}}} \right\|^{ 2} }}{{{\text{E}}\left[ {\left\| {{\mathbf{h}}^{H} {\mathbf{cS}}} \right\|} \right]^{2} }}$$

The objective is to maximize SINR subjected to the constraint ||s||^2^ ≤ 1.

### An orthogonal adaptive optimization algorithm

The design of extended target based waveform is different from the design of other types of waveform. It requires the prior information of clutter and target statistics. The transmitted waveform needs to adapt to the changing environment in real time scenario. The clutter information is estimated by the received signals before the target appears. The information is collected from K secondary data. The aim is to design a waveform which is best suited for the detection of the target of interest.

The orthogonal waveforms have better correlation properties which are critical to reduce mutual interference and to increase range resolution. The adaptive waveforms have the capacity to mitigate clutter statistics and increase the detection capabilities. The orthogonal adaptive (optimal) waveform is developed from the proposed adaptive algorithm. The orthogonal adaptive (optimal) waveforms have better probability of detection and better resolution. This proposed algorithm guarantees the improved SINR.

The technique applied here is to optimize the filter based on the covariance matrix of clutter and noise. The target statistics and waveform (orthogonal waveform initially) are also considered. The covariance matrix is a trained matrix of clutter statistics for K secondary data. Here, the clutter information is estimated by the received signals before the target appears. The covariance matrix of filter and clutter statistics are estimated. Using this covariance matrix, the signal covariance matrix is estimated from target, noise, clutter and filter covariance matrix. Then this waveform covariance matrix is normalized and transmitted by NxR MIMO radar system. Thus, obtained waveform is orthogonal optimal waveform.

The objective is to maximize SINR subjected to the constraint ||**S**||^2^ ≤ 1 and to optimize by first solving **h** in terms of **S** (Pillai et al. 2000). The optimization problem becomes.

11 $$\mathop {\text{Max}}\limits_{{\mathbf{h}}} \frac{{\left\| {{\mathbf{h}}^{H} {\mathbf{\alpha S}}} \right\|^{2} }}{{{\mathbf{h}}^{H} {\text{E}}\left[ {{\mathbf{C}}\,{\mathbf{S}}\,{\mathbf{S}}^{H} {\mathbf{C}}^{H} } \right]\,{\mathbf{h}} + {\mathbf{h}}^{H} {\text{E}}\left[ {{\mathbf{n}}\,{\mathbf{n}}^{H} } \right]{\mathbf{h}}}}\,$$

${\mathbf{R}}_{\text{c,s}} \mathop = \limits^{\Delta } E\left[ {{\mathbf{C}}\,{\mathbf{S}}\,{\mathbf{S}}^{H} \,{\mathbf{C}}^{H} } \right]\quad {\text{and}}\quad {\mathbf{R}}_{n} \mathop = \limits^{\Delta } E\left[ {{\mathbf{n}}\,{\mathbf{n}}^{H} } \right]$ are estimated from clutter covariance matrix in (7) and noise covariance matrix in (8). The maximization of **h** is possible by minimizing $\mathop {\hbox{min} }\limits_{{\mathbf{h}}} {\mathbf{h}}^{H} \left( {{\mathbf{R}}_{\text{c,s}} + {\mathbf{R}}_{\text{n}} } \right){\mathbf{h}}$ such that ${\mathbf{h}}^{H} {\mathbf{\alpha S}} = 1$.

The solution to this is (Capon 1969)

12 $${\text{h}} = \mu \left( {{\mathbf{R}}_{\text{c,s}} + {\mathbf{R}}_{\text{n}} } \right)^{ - 1} {\mathbf{\alpha S}}$$

where, $\mu$ is a scalar which satisfies the equality constraint. This term can be neglected as it has no effect on the objective function.

The objective function now becomes ${\mathbf{S}}^{\text{H}} {\mathbf{T}}^{\text{H}} \left( {{\mathbf{R}}_{\text{c,s}} + {\mathbf{R}}_{\text{n}} } \right)^{ - 1} {\mathbf{TS}}$ which is a function of **S** only.

13 $$\mathop {\text{Max}}\limits_{\text{S}} E\left[ {{\mathbf{S}}^{\text{H}} {\varvec{\upalpha}}^{\text{H}} \left( {{\mathbf{R}}_{\text{c,s}} + {\mathbf{R}}_{\text{n}} } \right)^{ - 1} {\mathbf{\alpha S}}} \right]$$

Subjected to ||**s**||^2^ ≤ 1.

**Fig. 1:**
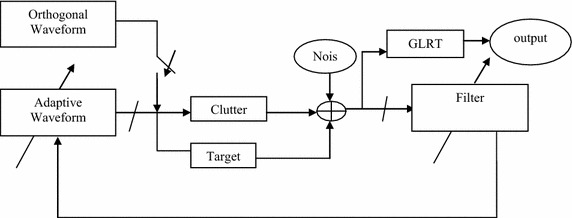
Block diagram of adaptive waveform generation

The adaptive algorithm is discussed below:

Step 1 Initialization
  The transmitting matrix of the DFCW waveforms as shown in Eq. (14) is modeled by optimized code set sequences using M_ACO optimization algorithm(Reddy and Uttarakumari 2014) (not in the scope of this paper). The objective function Eq. (4) is considered to minimize ASP and CP values. **S** is a matrix of (MxN).
  14 $${\mathbf{S}}\left( {\text{M,N}} \right) = \left[ {\begin{array}{*{20}c} {\text{a}_{{\text{11}}} } & {\text{a}_{{\text{12}}} } & \ldots & {\text{a}_{{\text{1N}}} } \\ {\text{a}_{{\text{21}}} } & {\text{a}_{{\text{22}}} } & \ldots & {\text{a}_{{\text{2N}}} } \\ \ldots & {} & {} & {} \\ \ldots & {} & {} & {} \\ {\text{a}_{{\text{M1}}} } & {\text{a}_{{\text{M2}}} } & \ldots & {\text{a}_{{\text{MN}}} } \\ \end{array} } \right]$$
  The extended target matrix is given by
  15 $${\varvec{\upalpha}} = \left[ {\begin{array}{*{20}c} {{\varvec{\upalpha}}\left( 0 \right)} & 0 & \ldots & 0 \\ {{\varvec{\upalpha}}\left( 1 \right)} & {{\varvec{\upalpha}}\left( 0 \right)} & \ldots & 0 \\ \ldots & {{\varvec{\upalpha}}\left( 1 \right)} & \ldots & 0 \\ {{\varvec{\upalpha}}\left( {\text{N}} \right)} & \ldots & \ldots & {{\varvec{\upalpha}}\left( 0 \right)} \\ 0 & {{\varvec{\upalpha}}\left( {\text{N}} \right)} & \ldots & {{\varvec{\upalpha}}\left( 1 \right)} \\ \ldots & \ldots & \ldots & {{\varvec{\upalpha}}\left( {\text{N}} \right)} \\ \end{array} } \right]$$
  Clutter covariance of size (1 × N) is estimated using the Eq. (16)
  16 $${\mathbf{r}}_{\text{ci}} = \frac{\text{M}}{\text{N}}\sum\limits_{{{\text{k}} = 1}}^{\text{K}} {\frac{{{\mathbf{r}}_{\text{ik}} {\mathbf{r}}_{\text{ik}}^{\text{H}} }}{{{\mathbf{r}}_{\text{ik}}^{\text{H}} {\mathbf{r}}_{\text{ik}} }}} ,\quad {\text{i}} = 1 \ldots M$$
  where, K is the number of secondary data, N is the length of the code set sequence of the waveform, **r**_ik_ is the received signal from the primary and secondary data. The clutter covariance matrix of size (M × N) is estimated using Eq. (16) and is as shown in Eq. (17)
  17 $${\mathbf{R}}_{\text{c}} = \left[ {\begin{array}{*{20}c} {{\mathbf{r}}_{\text{c1}} \left( 0 \right)} & {{\mathbf{r}}_{\text{c1}} \left( 1 \right)} & \ldots & {{\mathbf{r}}_{\text{c1}} \left( N \right)} \\ {{\mathbf{r}}_{\text{c2}} \left( 0 \right)} & {{\mathbf{r}}_{\text{c2}} \left( 1 \right)} & \ldots & {{\mathbf{r}}_{\text{c2}} \left( N \right)} \\ \ldots & {} & {} & {} \\ {{\mathbf{r}}_{\text{cM}} \left( 0 \right)} & {{\mathbf{r}}_{\text{cM}} \left( 0 \right)} & \ldots & {{\mathbf{r}}_{\text{cM}} \left( N \right)} \\ \end{array} } \right]$$
Step 2 Training of waveform and filter
  The filter coefficients are trained based on the covariance matrix of clutter and noise. The R_c,s_ is obtained by using clutter covariance matrix given in Eq. (18) and transmitting matrix using Eq. (14) for DFCW waveforms.
  18 $${\mathbf{R}}_{cs} = E\left[ {{\mathbf{R}}_{c} {\mathbf{S}}\,{\mathbf{S}}^{H} {\mathbf{R}}_{c}^{H} } \right]$$
  19 $${\mathbf{h}} = \left( {{\mathbf{R}}_{cs} + {\mathbf{R}}_{n} } \right)^{ - 1} {\mathbf{\alpha S}}$$
  The transmitting waveform is adapted to the dynamic environment using Eqs. (20) and (21). Using Eq. (22), the waveform is normalized. The covariance matrix of clutter and filter is estimated using Eq. (20) and finally, the waveform matrix is estimated using Eq. (22). Thus, obtained waveforms are orthogonal adaptive waveforms developed using adaptive algorithm.
  20 $${\mathbf{R}}_{ch} = E\left[ {{\mathbf{C}}^{H} {\mathbf{hh}}^{H} {\mathbf{C}}} \right]$$
  21 $${\mathbf{S}} = \left( {{\mathbf{R}}_{ch} + {\mathbf{h}}^{H} {\mathbf{R}}_{n} {\mathbf{h}}} \right)^{ - 1} {\varvec{\upalpha}}^{H} {\mathbf{h}}$$
  22 $${\mathbf{S}} = \frac{{\mathbf{S}}}{{\left\| {\mathbf{S}} \right\|}}$$
Step 3 The obtained orthogonal adaptive waveform (S) is substituted in Eq. (10) to obtain SINR, SCR and SNR values. The S matrix and values of SINR, SCR and SNR are noted and step 2 is repeated. Out of these two values, the one with highest CF waveforms is noted. The process is repeated for 100 simulations.
  This adaptive algorithm has orthogonally optimized DFCW_LFM waveforms with the prior knowledge of the channel and clutter, i.e., environment. The results of this algorithm are better than the iterative algorithm (Chen and al 2009) due to the collection of the secondary data for K samples. Using Eq. (17), the filter is initially trained for clutter statistics without target statistics.
  Figure 1 illustrates the adaptive algorithm to generate adaptive waveform. Initially the orthogonal waveforms are generated using optimization algorithm (Reddy and Uttarakumari 2014) (not in the scope of this paper) and then transmitted through the channel. The performance at the receiver degrades due to the clutter. Hence to increase the performance, the waveforms are modified based on the clutter and target statistics and also the filter coefficients are adapted based on the covariance matrix of clutter, target statistics and waveforms. The waveforms are orthogonally optimized based on the covariance matrix of noise, clutter and filter with target statistics. The adaptive algorithm adapts the waveform to the rapidly changing environment by increasing the SINR, SCR and SNR values.

### GLRT

In low resolution maritime radar system, the model of clutter is a complex Gaussian process. As the radar resolution increases, the clutter no longer acts as a Gaussian model and it can be described as a non-Gaussian clutter model for heavy-tailed clutter distributions. Using maximum likelihood estimation (MLE) method, the unknown parameters like the clutter power level and RCS of the target are estimated. To cancel the clutter and make the detector fully adaptive, the primary data covariance matrix is assumed to be known initially. Then, the secondary data covariance matrix is derived and placed in place of covariance matrix. Thus, the dynamic decision-based detector, Generalized GLRT detector (Cui et al. 2010) is developed. The GLRT detector shows excellent detection performance against the compound Gaussian clutter for high resolution MIMO radars. The clutter has exponential correction structure covariance matrix Ro, the (i,j) element of which is ρ^|i−j|^, here ρ is the one-lag correlation coefficient. The Power Spectral Density of clutter is generally located in low frequency region and Clutter spread is controlled by v. v is the parameter ruling the shape of the distribution. To analyze the probability of detection with orthogonal and adaptive waveforms, the parameters considered are P_fa_ = 10^−4^, N = 4, M_T_ = 4, M_R_ = 4, ρ = 0.9, K = 32 and v = 0.5. The GLRT & Gaussian clutter GLRT (GC-GLRT) detectors are used to check the performance of orthogonal optimal waveforms in terms of probability of detection when SCR is low.

## Results

The simulation was carried out in MATLAB for 4 × 4 MIMO for extended target. 4 sets of orthogonal DFCW_LFM codes were generated using Modified Ant Colony Optimization (M_ACO) algorithm for sequence length of 4 (Reddy and Uttarakumari 2014). The orthogonal waveforms are then optimized using adaptive algorithm as explained in section III. The PSLR and ISLR are evaluated for each sequence and are tabulated along with the sequences in Table 2. The autocorrelation sidelobe peak (ASP) and crosscorrelation peak (CP) for the corresponding waveforms are shown in Table 3 in presence of compound Gaussian clutter and extended target. The diagonal terms show the normalized ASP or PSLR of the autocorrelation function. The off diagonal terms are the normalized (with respect to sequence length) max CP of the crosscorrelation function. The average ASP and CP are −13.80 dB (0.2043) and −33.34 dB (0.02154), respectively, for the sequence in presence of compound Gaussian clutter and extended target. These results show that the waveforms are also orthogonal with better autocorrelation and crosscorrelation when clutter is very spiky. The correlation properties are very important to best suit best for MIMO applications. Low crosscorrelation sidelobe levels are very critical for reducing mutual interference maximizing independent information and also to facilitate high range resolution. In Table 4, the average ASP and CP values are tabulated for orthogonal, iterative and adaptive waveforms.

**Table 2:** 4 sequences generated using adaptive algorithm with compound Gaussian clutter and extended target (N = 4 and M = 4)

Code set	Orthogonal optimized sequence	PSLR	ISLR
1	0.0469 − 0.0240i, 0.0215 − 0.0105i, −0.0224 + 0.0051i, 0.6849 + 0.0220i	−13.82	−20.97
2	−0.0014 + 0.0193i, −0.0008 + 0.0087i, 0.0027 − 0.0080i, −0.1376 + 0.2110i	−13.82	−21.04
3	−0.0122 + 0.0024i, −0.0055 + 0.0010i, 0.0054 + 0.0003i, −0.1538 − 0.0494i	−13.83	−21.04
4	−0.0055 + 0.0502i, −0.0029 + 0.0228i, 0.0077 − 0.0206i, −0.3793 + 0.5374i	−13.83	−20.99

**Table 3:** ASP and CP for M = 4 and N = 4 code using adaptive algorithm with compound Gaussian clutter and extended

Code set	1	2	3	4
1	0.2036	0.0249	0.0159	0.0649
2	0.0249	0.2035	0.0059	0.0239
3	0.0159	0.0059	0.2034	0.0153
4	0.0649	0.0239	0.0153	0.2037

**Table 4:** average value of ASP and CP values for orthogonal, itterative and adaptive waveforms

	ASP (in dB)	CP (in dB)
Orthogonal	−5.0876	−28.4962
Itterative (Chen and Vaidyanathan 2009)	−11.32	−28.0023
Adaptive	−13.79	−32.0065

These results clearly show an improvement in ASP and CP values when compared with orthogonal and iterative method. There is a drastic reduction in sidelobes and also waveforms are more uncorrelated in presence of compound Gaussian clutter and extended target.

**Fig. 2:**
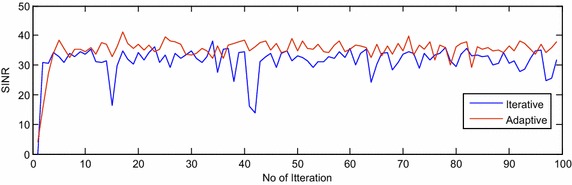
The SINR plot of adaptive and iterative algorithm for extended target in clutter

**Fig. 3:**
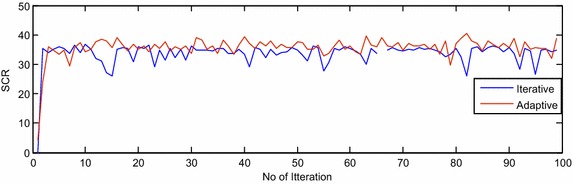
The SCR plot of adaptive and iterative algorithm for extended target in clutter

**Fig. 4:**
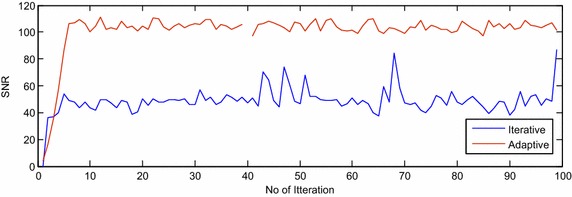
The SNR plot of adaptive and iterative algorithm for extended target in clutter

The proposed adaptive algorithm is compared with the existing iterative algorithm (Chen and al 2009) based on SINR, SCR and SNR performance measures for extended target model and are shown in Figs. 2, 3 and 4, respectively, in presence of compound Gaussian clutter. The oscillations in Figs. 2, 3 and 4 are due to the random behavior of compound Gaussian clutter and extended target scatterings. Improvement by 3, 4 and 25 dB are observed in SINR, SCR and SNR, respectively, using adaptive algorithms over iterative method (Chen and al 2009) in presence of compound Gaussian clutter and with extended target.

From Table 5, it can be concluded that the SINR, SNR and SCR values of orthogonally optimal waveforms generated using adaptive algorithm is better than the iterative algorithm in presence of compound Gaussian clutter and extended target.

**Table 5:** Maximum value of SINR, SCR and SNR for adaptive and iterative algorithm for extended target in clutter

	Adaptive (in dB)	Iterative (in dB) (Chen and Vaidyanathan 2009)
SINR	41.16	38.00
SCR	40.62	36.54
SNR	111.3	87.03

**Fig. 5:**
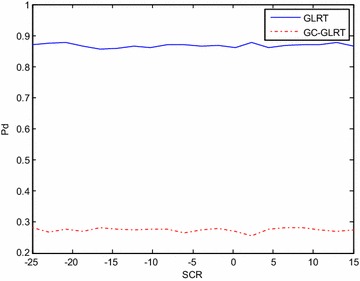
The plot of pd vs SCR for GC-GLRT and GLRT, for Pfa = 10^−4^, N = 4, Tx = 4, ρ = 0.9, Rx = 4, v = 0.5, K = 32 for* different waveforms* for adaptive

**Fig. 6:**
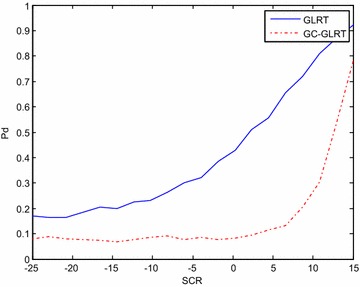
The plot of pd vs SCR for GC-GLRT and GLRT, for Pfa = 10^−4^, N = 4, Tx = 4, ρ = 0.9, Rx = 4, v = 0.5, K = 32 for* different waveforms* for iterative

The orthogonal adaptive waveforms generated by adaptive algorithm in presence of clutter and extended target are subjected to GLRT and GC-GLRT (Cui et al. 2010) to check the performance of these waveforms in terms of probability of detection when SCR is low. The waveform developed by adaptive algorithm show better detection performance even for lower SCR when GLRT and GC-GLRT (Cui et al. 2010) is adapted and is clearly shown in Figs. 5 and 6. So, this algorithm shows better result compared to iterative for lower SCR also.

## Conclusion

The adaptive algorithm develops waveforms that are orthogonal and exhibits good correlation properties and also shows good detection at lower SCR when adapted for GLRT. The numerical results show that the proposed adaptive algorithm has better SINR, SNR and SCR performance compared to the existing iterative algorithm.

## References

[CR2] Bo Z, Zhen D, Nong LD (2011). Design and performance analysis of orthogonal coding signal in MIMO-SAR. Sci China Inf Sci.

[CR3] Capon J (1969) High-resolution frequency-wavenumber spectrum analysis. In: IEEE Proceedings, vol. 57, no. 8, pp. 1408–1418

[CR4] Chen CY, Vaidyanathan PP et al (2009) MIMO radar waveform optimization with prior information of the extended target and clutter. IEEE Trans Signal Proc 57(9):3533–3544

[CR5] Cui G, Kong L, Yang X (2010). Two-step GLRT design of MIMO radar in compound-gaussian clutter. IEEE Radar Conf.

[CR6] Deng H (2004). Discrete frequency-coding waveform design for netted radar systems. IEEE Signal Process Lett.

[CR7] Fay FA, Clarke J, Peters RS (1997) Weibull distribution applied to sea clutter. In: Proceedings of IEEE Radar conference. London, pp. 101–103

[CR8] Friedlander B (2006). Adaptive waveform design for a multi-antenna radar system. Fortieth Proc Asilomar Control Signals Syst Comput.

[CR9] Gini F, Farina A, Montanari M (2002) Vector subspace detection in compound-Gaussian clutter, part-II: performance analysis. In: IEEE transaction on Aerospace Electronic system, vol. 38, no. 4, pp. 1312–1323

[CR10] Haimovich AM, Blum RS, Cimini LJ (2008). MIMO Radar with widely separated antennas. IEEE Signal Process Mag.

[CR11] Hu J, Tung W, Gao JB (2006) Modeling sea clutter as a nonstationary and non extensive random process. In: Proceedings of IEEE Radar Conference. Verona

[CR12] Jakeman E, Pusey PN (1976) A model for non- Rayleigh sea echo. In: IEEE Transactions on Antennas Propagation, vol. AP-24, no. 6, pp. 806–814

[CR13] Li J, Stoica P (2007). MIMO Radar with Colocated Antennas. IEEE Signal Process Mag.

[CR14] Liu B, He Z, He O (2007). Optimization of orthogonal discrete frequency-coding waveform based on modified genetic algorithm for MIMO radar. Int Conf Commun Circuit Syst.

[CR15] Liu B, He Z, Li J et al (2008) Mitigation of autocorrelation sidelobe peaks of orthogonal discrete frequency-coding waveform for MIMO radar. Proc IEEE Radar Conf:1–6. doi:10.1109/RADAR.2008.4720797

[CR16] Nijsure Y, Chen Y, Yuen C (2013). Adaptive distributed MIMO radar waveform optimization based on mutual information. IEEE Trans Aerosp Electr Syst.

[CR17] Pillai SU, Oh HS, Youla DC, Guerci JR (2000) Optimal transmit receiver design in the presence of signal-dependent interference and channel noise. IEEE Transaction on Information Theory, vol. 46, no. 2, pp. 577–584

[CR18] Reddy BR, Uttarakumari M (2014) Optimization of discrete frequency coding waveform for MIMO radar using modified ant colony optimization algorithm. In: First International Conference on Netwroks & Soft Computing (ICNSC), pp. 15–19. doi: 10.1109/CNSC.2014.6906667

[CR19] Sen S and Glover CW (2012)Adaptive waveform design based on multi-objective optimization for OFDM-STAP radar. In: Proceedings of International Conference on Engineering Optimization. Rio de Janeiro, pp. 1–10

[CR1] Sen S et al (2013) Designing OFDM radar waveform for target detection using multi-objective optimization. In: Advances in heurisitc signal process & applications. pp. 35–61. doi:10.1007/978-3-642-37880-5_3

[CR20] Trunk GV(1973) Radar properties of Non-Rayleigh sea clutter. In: IEEE Transactions on Aerospace and Electronic Systems, vol. AES-9, issue 1

[CR21] Ward KD (1981). Compound representation of high resolution sea clutter. IET Trans Electron Lett.

[CR22] Zhang JD, Zhu XH, Wang HQ (2009). Adaptive radar phase-coded waveform design. IET Electron Lett.

